# Supplementary material for: Polyketide synthase gene clusters in the ericoid mycorrhizal fungus Oidiodendron maius: genome-wide prediction, expression profiling, and functional characterization of knockout mutants
Source: Front Plant Sci. 2026 Sep 16;17:1924430. doi: 10.3389/fpls.2026.1924430 (PMC13623903; doi:10.3389/fpls.2026.1924430)
Supplement: Supplementary file 1 [file SupplementaryFile1.zip › Supplementary Materials/Supplementary Folder 1.zip!/Oidma1_AssemblyScaffolds_Repeatmasked/knownclusterblast/cluster33/jgi.p_Oidma1_169139_mibig_hits.html]

| MIBiG Protein | Description | MIBiG Cluster | MiBiG Product | % ID | % Coverage | BLAST Score | E-value |
| --- | --- | --- | --- | --- | --- | --- | --- |
| AGA37274.1 | negative\_regulator | BGC0000819 | Paraherquamide\_biosynthetic\_gene\_cluster | 25 | 83.5294117647 | 73 | 6e-13 |
| ADM34134.1 | NmrA\_family\_protein | BGC0001084 | Notoamide\_/\_stephacidin\_biosynthetic\_gene\_cluster | 35 | 43.2352941176 | 71 | 2.3e-12 |
| XP\_002384043.1 | NmrA-like\_family\_protein | BGC0001234 | 4,4'-piperazine-2,5-diyldimethyl-bis-phenol\_biosynthetic\_gene | 30 | 48.2352941176 | 66 | 5.6e-11 |
| ACY06277.1 | hypothetical\_protein | BGC0001042 | Sanglifehrin\_A\_biosynthetic\_gene\_cluster | 25 | 66.1764705882 | 64 | 3.7e-10 |
| XP\_002384854.1 | hscarg\_dehydrogenase,\_putative | BGC0001234\_c2 | 4,4'-piperazine-2,5-diyldimethyl-bis-phenol\_biosynthetic\_gene | 32 | 47.3529411765 | 57 | 2.6e-08 |
